# Heart Failure with Preserved Ejection Fraction: An Urgent Need for Precision Medicine

**DOI:** 10.3390/jcm10091801

**Published:** 2021-04-21

**Authors:** Gema Miñana, Julio Núñez

**Affiliations:** 1 Cardiology Department and Heart Failure Unit, Hospital Clínico Universitario de Valencia, INCLIVA, 46010 Valencia, Spain; gemineta@gmail.com; 2 Medicine Department, Universitat de Valencia, 46010 Valencia, Spain; 3 CIBER Cardiovascular, 28029 Madrid, Spain

Heart failure with preserved (HFpEF) and mid-range ejection fraction (HFmrEF) constitute two heart failure categories, representing about 50–70% of the total [1]. For both of these heart failure subgroups, there are substantial gaps in our understanding of their pathophysiology, diagnosis, clinical course, and optimal management, among others. This classification, based on left ventricular ejection fraction, reflects a pragmatic but simplistic view of heart failure syndromes’ complexity, particularly those with HFpEF. In both situations, we urgently require better pathophysiological and clinical phenotyping. Only moving beyond this left ventricular systolic function classification and considering other cardiac and non-cardiac parameters will allow us to more accurately evaluate different management strategies. Now, more than ever, we need to leave behind the old postulates focused on traditional views of the heart failure syndromes and advance in “precision medicine”.

In this Special Issue of the *Journal of Clinical Medicine*, we present seven works (two reviews and five original articles) addressing different and relevant aspects of HFpEF and HFmrEF. In the first review, Bayés-Genís and collaborators focus on the non-left ventricular mechanisms that play a crucial role in the transition from pre-HFpEF to symptomatic HFpEF. In this work, the authors discuss the role of the atria, right heart cavities, kidneys, and systemic inflammation on this transition [2]. On the other hand, in an elegant manuscript, Koufou EE et al. reviewed the evidence endorsing different treatment strategies in patients with HFmrEF, including pharmacological and non-pharmacological therapies [3]. In this review, the authors rightly questioned whether LVEF should continue to guide HF-therapeutic approaches, postulating alternative methods that potentially yield a better characterization and selection of the right treatment strategy [3].

In this issue, we also have the opportunity to present five original articles addressing important clinical aspects in HFpEF. First, Rettl R. et al. aimed to evaluate the proportion of patients who participated in a prospective national registry that fulfills the PARAGON-HF trial’s eligibility criteria [4]. These studies are relevant since they question whether findings from recent randomized clinical trials in HFpEF can easily be extrapolated to daily clinical practice. 

In another article, Santas et al. point out the prognostic importance of right-heart failure in HFpEF in 1355 patients consecutively discharged for decompensated HFpEF. These authors propose a right-ventricular failure staging system by combining the tricuspid annular plane systolic excursion to pulmonary artery systolic pressure ratio with functional tricuspid regurgitation severity [5]. In another study, Cho JH et al. evaluated the predictive utility of neutrophil to lymphocytes ratio, a simple and widely available marker, for risk stratification in a large Korean registry of patients with acute heart failure [6]. Considering the potential and the emerging role of inflammation in HFpEF, a specific analysis in HFpEF is also provided. 

Another work published in this issue focuses on the clinical relevance of iron deficiency in determining the functional capacity and quality of life of HFpEF patients. With this purpose in mind, Alcaide-Aldeano A et al. analyzed different surrogate biomarkers of iron deficiency in 447 HFpEF patients [7]. Finally, Balogh Z et al., in a non-randomized-design, investigated the long-term effects of endoscopic mitral valve repair on clinical outcomes in a cohort of HFpEF and atrial functional mitral regurgitation compared to the standard of care [8].
